# The Placental Microbiome Varies in Association with Low Birth Weight in Full-Term Neonates

**DOI:** 10.3390/nu7085315

**Published:** 2015-08-17

**Authors:** Jia Zheng, Xinhua Xiao, Qian Zhang, Lili Mao, Miao Yu, Jianping Xu

**Affiliations:** Department of Endocrinology, Key Laboratory of Endocrinology, Ministry of Health, Peking Union Medical College Hospital, Diabetes Research Center of Chinese Academy of Medical Sciences & Peking Union Medical College, Beijing 100730, China; E-Mails: zhengjiapumc@163.com (J.Z.); rubiacordifolia@yahoo.com (Q.Z.); maolili912@163.com (L.M.); yumiao@medmail.com.cn (M.Y.); jpxuxh@163.com (J.X.)

**Keywords:** low birth weight, fetal programming hypothesis, placenta, microbiome, full-term birth

## Abstract

Substantial evidence indicated that low birth weight was an independent risk factor for obesity, impaired glucose regulation, and diabetes later in life. However, investigations into the association between low birth weight and placental microbiome in full-term neonates are limited. Placentas were collected from low birth weight (LBW) and normal birth weight (NBW) full-term neonates (gestational age 37 w0d–41 w6d) consecutively born at Peking Union Medical College Hospital. The anthropometric measurements were measured and 16S ribosomal DNAamplicon high-throughput sequencing were utilized to define bacteria within placenta tissues. It showed that birth weight, ponderal index, head circumference, and placenta weight were significantly lower in LBW than NBW neonates (*p <* 0.05). The operational taxonomic units (OTUs) (*p <* 0.05) and the estimators of community richness (Chao) indexes (*p <* 0.05) showed a significantly lower diversity in LBW than NBW neonates. There were significant variations in the composition of placenta microbiota between the LBW and NBW neonates at the phylum and genus level. Furthermore, it indicated that Lactobacillus percentage was positively associated with birth weight (*r =* 0.541, *p =* 0.025). In conclusion, our present study for the first time detected the relationship between birth weight and placental microbiome profile in full-term neonates. It is novel in showing that the placental microbiome varies in association with low birth weight in full-term neonates.

## 1. Introduction

Nowadays, the prevalence of diabetes is increasing extremely rapidly worldwide. However, the pathogenesis of diabetes has not been clearly understood yet. Traditionally it has been widely acknowledged that genes together with adult lifestyle factors including diet and exercise habits determine the risk of developing some chronic non-communicable diseases such as obesity, insulin resistance, and diabetes mellitus in later life. Recently, attention has been paid to distal risk factors in early life. There is substantial evidence that intrauterine environment plays a critical role in determining our susceptibility to these diseases in adulthood, which is known as “fetal programming hypothesis” [1]. The pioneering study by Hales *et al.* was the first to show that people with low birth weight could increase the susceptibility to type 2 diabetes later in life [2]. Numerous studies demonstrated that there was an inverse relationship between birth weight (as a marker of fetal growth) and impaired glucose tolerance, insulin resistance, and diabetes in adult life [3]. In our previous study, it further indicated that low birth weight was an independent risk factor for later impaired glucose tolerance, diabetes, and an increased presentation of metabolic syndrome components [4,5].

The human microbiome is an enormous community of microorganisms occupying the habitats of the human body. Different microbial communities are found in each of the varied environments of human anatomy. The aggregate microbial gene totally surpasses that of the human genome by orders of magnitude [6]. In 2007, the National Institutes of Health (NIH) initiated the Human Microbiome Project (HMP) which was focused on developing research resources to enable the study of the microbial communities that live in and on our bodies and the roles they play in human health and disease. Recently published works by the HMP Consortium have provided the first reliable estimates of the breadth of structure, function, and diversity of the healthy human microbiome across multiple body sites [7]. Most organs including external (stool, skin) or internal (mouth, gut, stomach, urogenital tract, lungs) appear to be associated with human health and disease [8]. Previous studies have indicated that gut microbiota comprises a total genome of approximately 150 times as big as the human genome [9] and it is important in regulating metabolic pathways in healthy people and in patients with obesity, diabetes, and cardiovascular diseases [10]. However, little information is known about the placental microbiome.

The placenta is a vital organ that connects the developing fetus to the uterine wall to allow nutrient uptake and gas exchange (via the mother’s blood supply), fight against internal infection, and produce hormones to support pregnancy [11]. For a long time, it was thought that the fetus developed in a sterile environment. However, contrary to the prevailing idea of a “sterile” intrauterine environment [12], a recent study demonstrated that the placenta harbors a unique microbiome, composed of nonpathogenic commensal microbiota. It also revealed associations of the placental microbiome with a remote history of antenatal infection, such as urinary tract infection and preterm birth [13]. Recent advances in 16S ribosomal DNA (rDNA) amplicon high-throughput sequencing could facilitate us to extensively define bacteria within placenta tissue. Another study indicated that term and preterm labor were associated with distinct microbial community structures in placental membranes which were independent of mode of delivery [14]. A recent study suggested that the preterm placental microbiome varies in association with excess maternal gestational weight gain [15]. However, investigations into the association between placental microbiome and birth weight are limited. Considering that birth weight is a marker of fetal growth and an important factor determining the susceptibility to metabolic diseases in adult life, our aim in this study was to investigate whether the placental microbiome varies in association with low birth weight in full-term neonates

## 2. Experimental Section

### 2.1. Ethics Statement

Informed written consent was obtained from all participants, and the study protocol was approved by the Institutional Review Board and Ethics Committee of Peking Union Medical College Hospital (S-002).

### 2.2. Participants

The study population consisted of 24 (12 low birth weight and 12 normal birth weight) full-term neonates (gestational age 37 w0d–41 w6d) consecutively born at Peking Union Medical College Hospital Birth weight was used to identify cases and controls: low birth weight (LBW) is <3000 g and normal birth weight (NBW) is ≥3000 g and <4000 g. The birth weight of <3000 g but not <2500 g was determined as the cut-off due to the fact that birth weight <2500 g neonates only took a very small proportion in our full-term subjects. The more important reason is that low birth weight (<3000 g) is also an independent risk factor for later impaired glucose tolerance and diabetes in previous studies [5,16]. All the neonates were spontaneous birth by vaginal delivery in order to prevent confounding factors related to the mode of delivery. Selected low birth weight cases and controls were matched for gestational age, race/ethnicity, body mass index (BMI) and age. Infants whose mothers had multifetation, chronic hypertension, pregnancy-induced hypertension, gestational diabetes mellitus, endocrine disorders, and other severe maternal illnesses, and infants with a gestational age <37 or >42 weeks, asphyxia at birth, congenital anomalies, and the presence of antepartum infections and antibiotic use (*i.e.*, sexually transmitted infection, urinary tract infection, skin abscesses, appendicitis, periodontal disease, or bacterial meningitis) were excluded from the study.The risk factors for low birth weight, such as smoking, drug use, poor maternal weight gain, prior low birth weight baby, little education, and low income were also excluded from the study.

### 2.3. Placental Samples

All samples were collected by trained pathology personnel according to the method previously described [13]. Briefly, following standard obstetrical practice, the placenta was delivered and immediately passed off to trained personnel in a sterile clean container. Then, four to six 1 cm × 1 cm × 1 cm cuboidal sections were circumferentially excised from separate areas of the placenta, each located 4 cm from the cord insertion site. The excision was performed by trained pathology personnel donning facial masks and sterile gloves and using a sterile scalpel and instruments. The placental surfaces including the maternal decidua and fetal chorion-amnion were thereafter excised and discarded to avoid potential contamination from maternal, fetal, or environmental surface exposure during delivery or at the time of specimen transport. Then, the placenta tissues were snap frozen in liquid nitrogen, and stored at −80 °C for further analysis.

### 2.4. Anthropometric Measurement

Data were collected at delivery according to our previous publication [17]. Three trained research nurses and assistants collected data on maternal, pregnancy, and birth characteristics using structured study questionnaires through face-to-face interviews and medical chart reviews. Demographic data included maternal medical history, maternal age, and BMI before pregnancy. The variables of the neonates included gestational age, birth weight, body length, ponderal index (PI), head circumference, and placenta weight. PI was calculated as body weight (kg)/body length (m)^3^. The body weight of each neonate and placenta weight were determined to the nearest 1 g using an electronic scale. Body length was determined to the nearest 0.1 cm in the supine position with a length board. Head circumference was determined with a plastic tape to the nearest 0.1 cm.

### 2.5. Microbial Diversity Analysis

#### 2.5.1. DNA Extraction and Polymerase Chain Reaction (PCR) Amplification

Microbial DNA was extracted from placental tissue (about 100–150 mg) using the E.Z.N.A.^®^ DNA Kit (Omega Bio-tek, Norcross, GA, USA). The V3-V4 region of the bacteria 16S ribosomal RNA gene were amplified by PCR using primers 338F 5′-ACTCCTACGGGAGGCAGCA-3′ and 806R 5′-GGACTACHVGGGTWTCTAAT-3′, where barcode is an N-base sequence (N represent a unique 6 to 8-nt barcode) unique to each sample. PCR reactions were performed in 20 μL mixture containing 4 μL of 5 ×FastPfu Buffer, 2 μL of 2.5 mM dNTPs, 0.8 μL of each primer (5 μM), 0.4 μL of FastPfu Polymerase, and 10 ng of template DNA. Because of the concern for bias in low-abundance samples, all specimens were thrice extracted and thrice run to enable triplicate extraction and sequencing. Sequences from all three extractions were combined for analysis, according to the method previously described [15].

#### 2.5.2. IlluminaMiSeq Sequencing and Bioinformatic Analysis

Amplicons were extracted from 2% agarose gels and purified using the AxyPrep DNA Gel Extraction Kit (Axygen Biosciences, Union City, CA, USA). Purified amplicons were pooled in equimolar and paired-end sequenced (2 × 300) on an IlluminaMiSeq platform according to the standard protocols. Raw fastq files were demultiplexed, quality-filtered using QIIME (version 1.17) with the following three criteria: (1) The 300 bp reads were truncated at any site receiving an average quality score <20 over a 10 bp sliding window, discarding the truncated reads that were shorter than 50 bp; (2) exact barcode matching, two nucleotide mismatch in primer matching, reads containing ambiguous characters were removed; (3) only sequences that overlap longer than 10 bp were assembled according to their overlap sequence. Reads which could not be assembled were discarded. Operational Units (OTUs) were clustered with 97% similarity cut-off using UPARSE (version 7.1) [18] and chimeric sequences were identified and removed using UCHIME. The phylogenetic affiliation of each 16S rRNA gene sequence was analyzed by RDP Classifier [19] against the silva (SSU115) 16S rRNA database using confidence threshold of 70% [20].

### 2.6. Statistical Analysis

The data were expressed as the mean ± standard deviation (S.D.). Statistical analyses of anthropometric measurement were performed with Student’s *t*-test. Unweighted UniFrac distance metrics analysis was performed using OTUs for each sample and PCoA plots based on unweighted Unifrac metrics. Metastats was used to compare the relative abundance of each taxon at different taxonomic levels between LBW and NBW neonates [21]. Correlation analyses between relative abundance of sequences belonging to different bacterial class and birth weight were performed by using Spearman’s correlation analyses. *p* value < 0.05 were considered to indicate statistical significance. All statistical analysis was calculated with SPSS 21.0 (SPSS, Inc., Chicago, IL, USA).

## 3. Results

### 3.1. Clinical Characteristics and Anthropometric Measurements

The clinical characteristics and anthropometric measurements were shown in Table 1. It indicated that birth weight (2623 ± 207 *vs.* 3452 ± 96, *p <* 0.01), ponderal index (23.2 ± 5.2 *vs.* 26.2 ± 1.4, *p =* 0.02), head circumference (32.9 ± 1.6 *vs.* 34.6 ± 0.8, *p =* 0.013) and placenta weight (492.8 ± 38.5 *vs.* 701.2 ± 106.7, *p <* 0.01) were significantly lower in LBW than NBW subjects. The birth weight of normal birth weight subjects in our present study is similar to the birth weight for the babies delivered at Peking Union Medical College Hospital in one previous study [17]. However, no significant differences were detected in maternal age, maternal BMI before pregnancy, gestational age, and body length between LBW and NBW neonates (Table 1).

### 3.2. Characteristics of Sequencing Results

A total of 403,965 high-quality sequences were produced in this study, with an average of 16,831 sequences per sample (Table 2). The Good’s coverage of each group was over 97%, indicating that the 16 S rDNA sequences identified in the groups represent the majority of bacteria present in the samples of this study. The operational taxonomic unit (OTU), the estimators of community richness (Chao) and diversity (Shannon) are also shown in Table 2. There were statistically significant differences of OUT (49 ± 4.18 *vs.* 48.44 ± 7.89, *p =* 0.026) and Chaoindexes (57 ± 7.19 *vs.* 54.44 ± 13.33, *p =* 0.044) between NBW and LBW groups, demonstrating the significantly lower richness found in LBW neonates compared to NBW neonates.

**Table 1:** Clinical characteristics and anthropometric measurements of fetus at birth.

Group	N (Male/Female)	Maternal Age (Years)	Maternal BMI before Pregnancy	Gestational Age (Weeks)	Birth Weight (g)	Body Length (cm)	Ponderal Index (kg/m^3^)	Head Circumference (cm)	Placenta Weight (g)
NBW	12 (6/6)	31 ± 3	21.3 ± 2.2	39.1 ± 0.8	3452 ± 96	50.8 ± 1.1	26.2 ± 1.4	34.6 ± 0.8	701.2 ± 106.7
LBW	12 (6/6)	31 ± 4	21.1 ± 2.9	38.9 ± 0.9	2623 ± 207	49.2 ± 4.6	23.2 ± 5.2	31.9 ± 1.6	492.8 ± 38.5
*p* value	-	0.76	0.82	0.58	<0.01 **	0.34	0.02 *	0.013 *	<0.01 **

Data reported as the mean ± Standard Deviation (SD). * *p* < 0.05, ** *p* < 0.01 *vs.* NBW. NBW, normal birth weight; LBW, low birth weight; BMI, body mass index.

**Table 2:** Sequencing data summary.

	NBW	LBW	*p* Value
Sequences	17,600 ± 3693	16,150 ± 3768	0.49
OTUs	49 ± 4.18	48.44 ± 7.89	0.026 *
Chao	57 ± 7.19	54.44 ± 13.33	0.044 *
Shannon	0.96 ± 0.12	1.06 ± 0.36	0.083

Data represents as mean ± S.D. (*n =* 12, in each group). Statistical analyses were performed with Student’s *t*-test between the two groups. The number of OTUs, richness estimator Chao, and diversity estimator Shannon were calculated at 3% distance. * *p <* 0.05 LBW vs the NBW group. Abbreviations: LBW, Low birth weight; NBW, Normal birth weight.

### 3.3. Principal Coordinates Analysis (PCA) between LBW and NBW Groups

**Figure 1:**
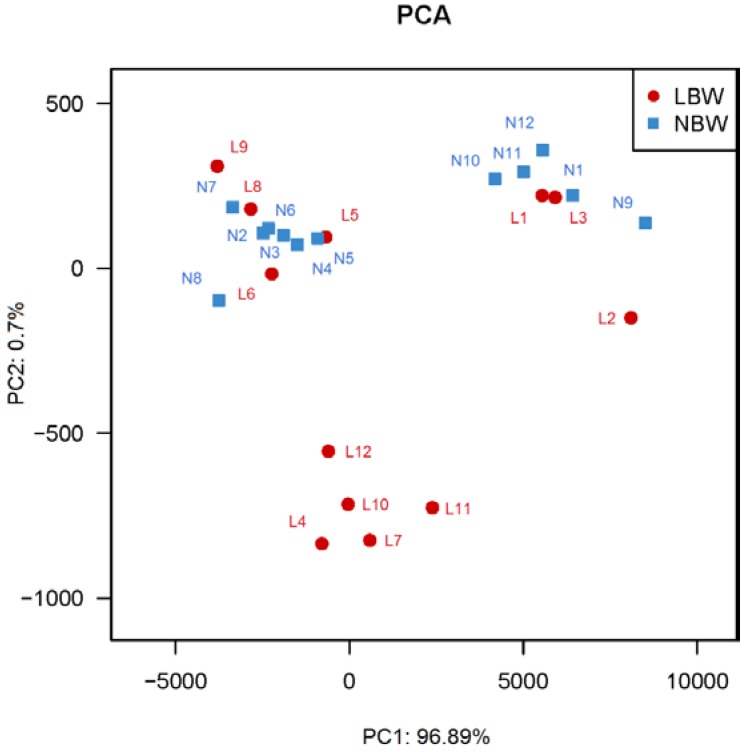
Principal Coordinate Analysis (PCA) plots in LBW and NBW neonates. PCA plots based on unweighted Unifrac metrics. *n =* 12, in each group. Abbreviations: LBW, Low birth weight; NBW, Normal birth weight.

Closer analysis of bacterial differences induced by birth weight were determined by sequencing the 16S rRNA encoding genes present in the placenta. PCA of IlluminaMiSeq amplicon data demonstrated significantly separate clustering of the placenta communities between LBW and NBW groups with principal component (PC1) percent variation explained = 96.89% and PC2 percent variation explained = 0.7%. It indicated that there was a statistically significant clustering by virtue of body weight (Figure 1).

### 3.4. Microbial Structures of the Placenta in LBW and NBW Groups

**Figure 2:**
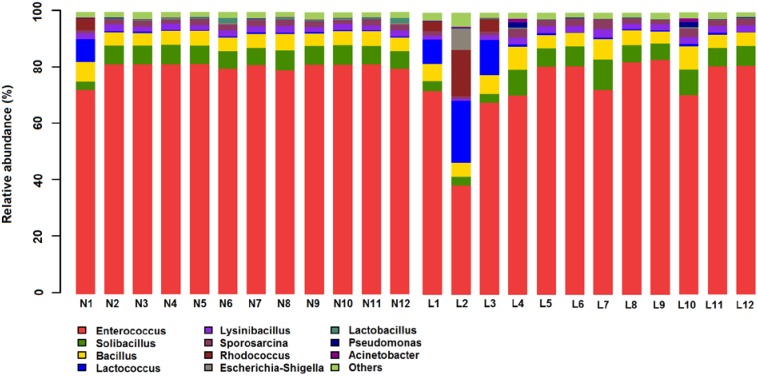
Relative abundance (%) of placenta microbiota in each sample at the genus level. *n =* 12, in each group. Abbreviations: LBW, Low birth weight; NBW, Normal birth weight.

**Figure 3:**
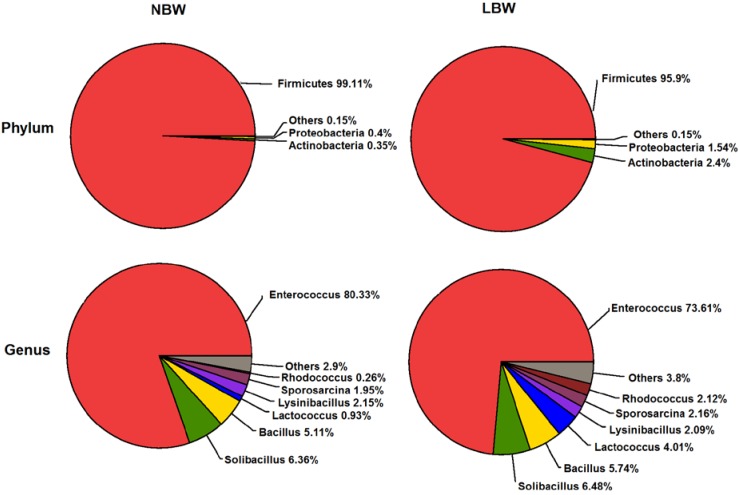
Differential placental microbial abundance plots at the phylum and genus level. *n =* 12, in each group. Abbreviations: LBW, Low birth weight; NBW, Normal birth weight.

**Figure 4:**
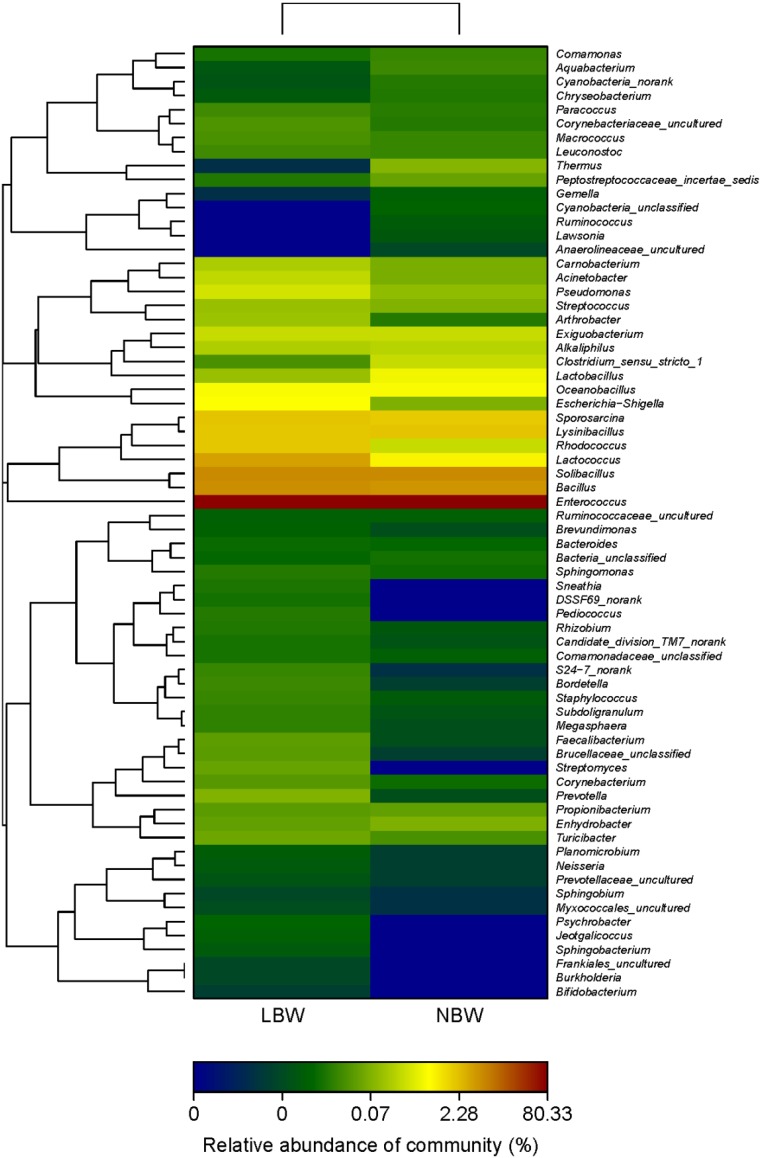
Heatmap analyses of abundant genera in each group. *n =* 12, in each group. The y axis is a neighbor-joining phylogenetic tree, each row is a different phylotype. The abundance plot shows the proportion of 16S rRNA gene sequences in each group. Abbreviations: LBW, Low birth weight; NBW, Normal birth weight.

Figure 2 shows relative abundance (%) of placenta microbiota in each sample at the genus level. The overall microbiota structure for each group at the phylum and genus level is shown in Figure 3. At the phylum level, the dominant phylum of the two groups was *Firmicutes*. The relative abundance (%) of *Firmicutes* was decreased in LBW group, while *Proteobacteria* and *Actinobacteria* were increased in LBW group, compared with NBW group. At the genus level, the dominant phylum of the two groups was *Enterococcus*. The relative abundance (%) of *Enterococcus* was decreased in LBW group, while *Lactococcus* and *Bacillus* were increased in LBW group, compared with NBW group. The relative abundance of microbiota sequences revealed that microbial structures of the placenta differed significantly between LBW and NBW groups. The heatmap according to bacterial genus level also demonstrated the same phenomenon (Figure 4).

### 3.5. Phylotypes Significantly Different between LBW and NBW Groups

To investigate whether the placental microbiome varies in association with low birth weight in full-term neonates, we compared the phylum- and genus-level relative abundance by body weight. As shown in Table 3, at the phylum level, *Fusobacteria* was significantly increased and *Cyanobacteria* was significantly decreased in LBW group, compared with NBW group (*p <* 0.05). The microbial composition was also significantly different at the genus level, with 13 significantly different genera between LBW and NBW groups (*p <* 0.05). It is indicated that the relative abundance (%) of *Lactobacillus*, *Clostridium_sensu_stricto_1*, *Cyanobacteria_unclassified*, *Ruminococcus*, *Lawsonia* and *Cyanobacteria_norank* were significantly lower in LBW group at the genus level. Conversely, the relative abundance (%) of *Megasphaera*, *Faecalibacterium*, *DSSF69_norank*, *Jeotgalicoccus*, *Pediococcus*, *Sneathia*, and *Sphingobacterium* were significantly higher in LBW group, compared with NBW group (Table 3).

**Table 3:** Phylotypes significantly different between LBW and NBW groups.

Taxonomic Rank		NBW (%)	LBW (%)	* *p* Value
phylum	Fusobacteria	0.000	0.011	0.001
phylum	Cyanobacteria	0.020	0.003	0.008
genus	Cyanobacteria_unclassified	0.007	0.000	0.001
genus	DSSF69_norank	0.000	0.013	0.001
genus	Jeotgalicoccus	0.000	0.005	0.001
genus	Pediococcus	0.000	0.017	0.001
genus	Sneathia	0.000	0.011	0.001
genus	Clostridium_sensu_stricto_1	0.253	0.026	0.002
genus	Lactobacillus	0.531	0.115	0.005
genus	Ruminococcus	0.004	0.000	0.007
genus	Megasphaera	0.003	0.016	0.008
genus	Faecalibacterium	0.002	0.038	0.014
genus	Lawsonia	0.004	0.000	0.014
genus	Sphingobacterium	0.000	0.007	0.016
genus	Cyanobacteria_norank	0.014	0.003	0.047

*n =* 12, in each group. Statistical analysis was performed by Metastats. Data of NBW and LBW groups were relative abundance (%) of all sequences in each group. * *p <* 0.05 LBW *vs.* NBW group. *p* value had no statistically significant difference (≥0.05) were not shown. Abbreviations: LBW, Low birth weight; NBW, Normal birth weight.

### 3.6. Variation in the Placental Microbiome Associated with Birth Weight

**Figure 5:**
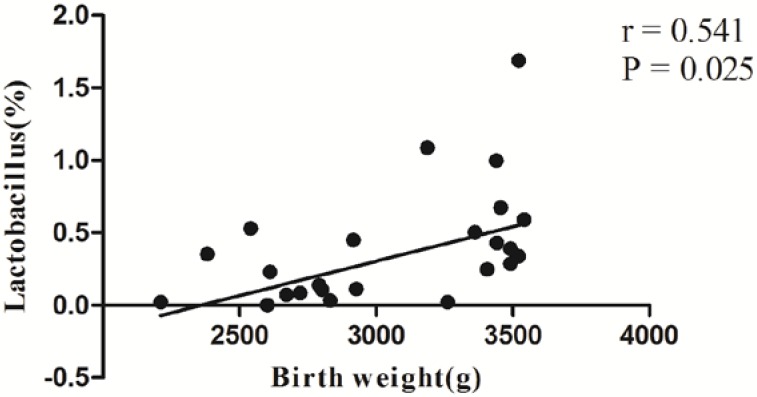
Correlation analyses between relative abundance (%) of sequences belonging to bacterial genus and birth weight. *n =* 24. Correlation analyses between relative abundance (%) of sequences belonging to a specific bacterial genus and birth weight were performed by using Spearman’s correlation analyses.

Correlation analyses between relative abundance (%) of sequences belonging to a specific bacterial genus and birth weight were performed by using Spearman’s correlation analyses. It is indicated that *Lactobacillus* percentage were positively associated with birth weight (*r =* 0.541, *p =* 0.025) (Figure 5).

## 4. Discussion

Substantial studies have shown that low birth weight can significantly increase the predisposition to the development of some metabolic diseases such as obesity and diabetes in adult life. Recently, numerous studies demonstrated that the microbiota could be considered as one major player in the development of obesity and diabetes mellitus [22,23]. Therefore, we investigated whether low birth weight was associated with significant variation in the placental microbiome profile. Consistent with previous studies, our study indicated that the placenta harbors a low-abundance microbiome. The placental microbiome was largely composed of nonpathogenic commensal microbiota from the Firmicutes, Actinobacteria, Proteobacteria, and Fusobacteria phyla in all the LBW and NBW neonates [13,15].

Our present study indicated that the placenta of LBW neonates had significantly lower bacterial richness and evenness than NBW neonates. In the gut, one large clinical study also demonstrated that individuals with a low bacterial richness are characterized by more marked overall adiposity, insulin resistance, and dyslipidaemia, which indicated that a low bacterial richness may be associated with abnormal glucose metabolism [24]. Similar to our study focused on placenta, one study previously demonstrated that decreased richness in the placenta is associated with spontaneous preterm birth [13]. Another study aimed to investigate whether the preterm placental microbiome varies by virtue of obesity or alternately by excess gestational weight gain and it indicated that preterm subjects with excess gestational weight gain also had decreased species richness. Thus, our study suggests that the decreased richness in placenta may be associated with birth weight in neonates.

Furthermore, 16S-based OTU analyses in our study revealed the relative abundance of Lactobacillus was negatively associated with birth weight. Most *Lactobacillus* in humans are considered un-harmful. Some strains of *Lactobacillus* may possess potential therapeutic properties including anti-inflammatory activities, as well as other features of interest. Several studies also showed that probiotic products could regulate blood glucose levels in diabetic human and *Lactobacilli* are often used as probiotic agents [25]. Naito *et al.* reported that oral administration of *Lactobacillus casei* strain Shirota had the potential to prevent obesity-associated metabolic abnormalities by improving insulin resistance in diet-induced obesity mice [26]. One study showed that DNA of *Lactobacillus rhamnosus* was detected in placenta samples independent of the mode of delivery. It also indicated that DNA from intestinal bacteria was found in most placenta samples and the horizontal transfer of bacterial DNA from mother to fetus may occur via placenta [27]. Therefore, it can be speculated that *Lactobacillus* may be the potential probiotic to modulate the placenta microbiota. However, more evidence and further investigation will be needed.

To our knowledge, our present study for the first time demonstrated the relationship between birth weight and placental microbiome profile in neonates. Strengths of this study also include that microbial sequences were isolated and identified successfully from placenta tissue which harbors a low-abundance microbiome. More important, the sterile sample collection and robust analysis were rigorously conducted. However, there are some limitations to our study. One primary limitation was the small sample size. Because low birth weight neonates only took a very small proportion in our study subjects which consisted of full-term and spontaneous birth by vaginal delivery neonates in order to minimize the effect of some confounding factors such as gestational age (full-term or preterm) and the mode of delivery (vaginal delivery or cesarean delivery). Our ongoing work is aimed to enlarge the sample size for further research. A second limitation was that our study just focused on comparative 16S rDNA analysis but not metagenomic studies which can provide additional insight into the function of complex microbial communities and their role in host health. Therefore, our further research will be focused on the function of microbial communities in placenta and their role in neonates and mothers. A third limitation was that due to the small sample size (*n =* 12), it has not been possible to perform a multivariate analysis, whose results are more reliable than those from univariate one. The fourth limitation was that the used definition of low birth weight (<3000 g) is not that universally accepted. This was due to the fact that birth weight <2500 g neonates only took a very small proportion in our full-term subjects. The more important reason is that low birth weight (<3000 g) is also an independent risk factor for later impaired glucose tolerance and diabetes in previous studies. However, our present study is also very meaningful which established an important theoretical foundation of metagenomic studies in placenta of different birth weight neonates. Another potential limitation to our study was the overall low abundance of microbiota in the placenta. However, we were able to overcome the limitations of low-abundance microbiome by multiple biological and technical replicates.

## 5. Conclusions

In conclusion, our present study is novel in showing that the placental microbiome varies in association with low birth weight in full-term neonates.
